# Cognitive Impairment Phenotypes in Patients with CKD Stages 3b and 4

**DOI:** 10.34067/KID.0000000000000474

**Published:** 2024-06-27

**Authors:** Mickaël Bobot, Julie Bruno, Stéphane Burtey

**Affiliations:** 1 Centre de Néphrologie et Transplantation Rénale, Hôpital de la Conception, AP-HM, Marseille, France; 2 Aix Marseille University, INSERM, INRAE, C2VN, Marseille, France; 3 CERIMED, Aix Marseille University, Marseille, France

**Keywords:** CKD, cognitive impairment, cognition, kidney-brain axis

Neurocognitive impairment is a frequent and underrecognized trouble in CKD, which has a major effect on the patients' quality of life and complicates their management throughout the evolution of their kidney disease. The severity of cognitive impairment is correlated to the severity of CKD.^1,2^ Moreover, an eGFR below 30 ml/min per 1.73 m^2^ seems as an independent risk factor of vascular dementia (hazard ratio, 2.56 [1.42–4.63], *P* = 0.002) or dementia from all causes (hazard ratio, 2.44 [1.79–3.34], *P* < 0.001).^3^ The prevalence of cognitive impairment can reach 30%–80% of patients with ESKD. In earlier stages of CKD, cognitive impairment is less studied, but reports suggest that cognitive impairment exists since CKD stage 3^4^ and seems to be more correlated to albuminuria than eGFR. In the French cohort CKD-Rein with mean eGFR of 33 ml/min per 1.73 m^2^, the eGFR is correlated to the risk of cognitive events during the 5 years of follow-up.^5^ Previous studies found that attention, orientation, and language were the most altered functions.^6^ Pathophysiology is only poorly understood. The classical risk factors of cognitive impairment like hypertension, diabetes mellitus, smoking, and dyslipidemia are highly prevalent in patients with CKD but cannot explain solely the frequency and severity of cognitive dysfunction during CKD.^7^ Growing experimental data suggest that uremic toxin accumulation could play a major role,^1^ notably indoxyl sulfate, known for its endothelial toxicity, leading to an increased permeability of the blood–brain barrier.^8^

In this issue of *Kidney360*, the study by Zhang *et al.* investigated cognitive impairment in 105 patients with CKD stage 3b and 4 from the Bicarbonate Administration in CKD Trial using the National Institutes of Health Toolbox Cognition battery.^9^ The cognitive tests were performed at the baseline study visit and evaluated many aspects of cognition: executive functions, working memory, episodic memory, cognitive processing speed, crystallized knowledge, and dexterity. The completion took about 45 minutes.

Overall, participants with CKD scored below the 50th percentile of the National Institutes of Health Toolbox reference population in all cognitive tests and dexterity; the more impaired functions were attention and dexterity, with approximately 30% of the patients with severe impairment (below the 16th percentile). Patients with CKD stage 4 had significantly lower scores in fluid cognition and working memory than patients with CKD stage 3b (*P* = 0.03 and *P* = 0.005, respectively).

Interestingly, this study highlights different phenotypes in cognitive impairment regarding sex in patients with CKD. Female patients displayed significantly more impairment in executive functions (on Flanker test, *P* = 0.006) but better performances in episodic memory and dexterity evaluation (*P* = 0.001 and *P* = 0.001, respectively).^9^

Thus, this study brings up interesting results in the field of cognitive impairment during CKD: Important cognitive dysfunction exists in earlier stages of CKD, and not only in patients with ESKD, and patterns CKD-associated differ between male and female patients.

One limitation of the study is its lack of a control group consisting patients without CKD matched on age, comorbidities, and treatments administered to patients. Indeed, medications frequently prescribed in patients with CKD could have an important effect on cognitive performance, notably anticholinergics drugs. Moreover, patients with CKD are more prone to have anemia and electrolyte disorders like hyponatremia or acidosis which have a negative effect on cognition during CKD as well. These electrolyte disorders can also be worsened by treatments like diuretics and or renin–angiotensin system inhibitors. While patients with metabolic acidosis and alkalosis were not included in this study, specific analyses regarding the type of medications taken, serum levels of sodium could be considered to assess the role of CKD *per se* on cognitive impairment.

The results emphasize the importance to improve our knowledge of how the kidney dysfunction is associated with impaired cognitive function. The onset of cognitive impairment occurs early in the life of patients with CKD. It is important to take it into account to improve and adapt care to patients with CKD. The attention dysfunction associated with CKD must encourage physicians to improve their skills for communication and to propose multidimensional information to better capture the attention of patients with CKD to improve the delivery of the information about kidney care and the adherence to treatments. One important effect of cognitive dysfunction could be the falls and the risk of fracture. Patients with CKD have an increased risk of falls, unexplained by classical risk factors. The addition of cognitive dysfunction in patients with eGFR below 40 ml/min per 1.73 m^2^ could potentially explain this increase.

This study paves the way for a better understanding of early cognitive dysfunction. It will be interesting to study, for example, the interrelationship between sleep and cognitive dysfunction in CKD because sleep disorders are frequent in CKD.^10^ It is well recognized that sleep disorders are associated with neurological disorders. The sleep defect observed in CKD could partially explain the cognitive impairment.

The kidney–brain axis is clearly a new field of research in kidney disease and could explain many complications associated with CKD. The research in this new field is critical for an improved care delivered to patients with CKD.

Attention should be raised on the screening for cognitive impairment in patients with CKD, even since stage 3b, and on the specificity of cognitive dysfunction phenotype in patients with CKD.

## Supplementary Material
