# Supplementary material for: Cognitive Impairment Phenotypes in Patients with CKD Stages 3b and 4
Source: Kidney360. 2024 Jun 27;5(6):787–8. doi: 10.34067/KID.0000000000000474 (PMC11219103; doi:10.34067/KID.0000000000000474)
Supplement: Supplementary file 1 [file kidney360-5-787-s001.pdf]

ASN American Society of Nephrology
JASN Journal of the American Society of Nephrology
KIDNEY360 Accessing Our World From Every Angle
CJASN Clinical Journal of the American Society of Nephrology

ASN Journal Disclosure Form

As per ASN journal policy, I have disclosed any financial relationship or commitment held by myself and/or my spouse/partner in the past 36 months as included below. I have listed my Current Employer below to indicate there is a relationship requiring disclosure. If no relationship exists, my Current Employer is not listed.

M. Bobot has nothing to disclose.

I understand that the information above will be published within the journal article, if accepted, and that failure to comply and/or to accurately and completely report the potential financial conflicts of interest could lead to the following: 1) Prior to publication, article rejection, or 2) Post-publication, sanctions ranging from, but not limited to, issuing a correction, reporting the inaccurate information to the authors' institution, banning authors from submitting work to ASN journals for varying lengths of time, and/or retraction of the published work.

Name: Mickaël Bobot
Manuscript ID: K360-2024-000277R1
Manuscript Title: Cognitive impairment phenotypes in patients with CKD stages 3b and 4
Date of Completion: April 15, 2024
Disclosure Updated Date: April 15, 2024

ASN
American Society of Nephrology
JASN
Journal of the American Society of Nephrology
KIDNEY360
Accessing Our World From Every Angle
CJASN
Clinical Journal of the American Society of Nephrology

ASN Journal Disclosure Form

As per ASN journal policy, I have disclosed any financial relationships or commitments I have held in the past 36 months as included below. I have listed my Current Employer below to indicate there is a relationship requiring disclosure. If no relationship exists, my Current Employer is not listed.

J. Bruno reports the following:
Employer: Assistance Publique - Hopitaux de Marseille; and Honoraria: Astra-Zeneca - Honoraria for a scientific presentation.

I understand that the information above will be published within the journal article, if accepted, and that failure to comply and/or to accurately and completely report the potential financial conflicts of interest could lead to the following: 1) Prior to publication, article rejection, or 2) Post-publication, sanctions ranging from, but not limited to, issuing a correction, reporting the inaccurate information to the authors’ institution, banning authors from submitting work to ASN journals for varying lengths of time, and/or retraction of the published work.

Name: Julie Bruno
Manuscript ID: K360-2024-000277R1
Manuscript Title: Cognitive impairment phenotypes in patients with CKD stages 3b and 4
Date of Completion: April 16, 2024
Disclosure Updated Date: April 16, 2024

ASN
American Society of Nephrology
JASN
Journal of the American Society of Nephrology
KIDNEY360
Accessing Our World From Every Angle
CJASN
Clinical Journal of the American Society of Nephrology

ASN Journal Disclosure Form

As per ASN journal policy, I have disclosed any financial relationships or commitments I have held in the past 36 months as included below. I have listed my Current Employer below to indicate there is a relationship requiring disclosure. If no relationship exists, my Current Employer is not listed.

S. Burtey reports the following:
Employer: aix-marseille université; Consultancy: Astra Zeneca; Lilly; Bayer; Alexion; CLS vifor, Amgen; Research Funding: DImerix; Astellas; Honoraria: fresenius kabi; Astra zeneca; boringer ingelheim, alexion, csl vifor; and Advisory or Leadership Role: CSL vifor.

I understand that the information above will be published within the journal article, if accepted, and that failure to comply and/or to accurately and completely report the potential financial conflicts of interest could lead to the following: 1) Prior to publication, article rejection, or 2) Post-publication, sanctions ranging from, but not limited to, issuing a correction, reporting the inaccurate information to the authors' institution, banning authors from submitting work to ASN journals for varying lengths of time, and/or retraction of the published work.

Name: Stephane Burtey
Manuscript ID: K360-2024-000277R1
Manuscript Title: Cognitive impairment phenotypes in patients with CKD stages 3b and 4")
Date of Completion: April 30, 2024
Disclosure Updated Date: April 30, 2024
